# Remdesivir treatment and clinical outcome in non-severe hospitalized COVID-19 patients: a propensity score matching multicenter Italian hospital experience

**DOI:** 10.1007/s00228-023-03499-z

**Published:** 2023-05-22

**Authors:** Emilio Attena, Alfredo Caturano, Anna Annunziata, Alberto Enrico Maraolo, Annunziata De Rosa, Francesco Maria Fusco, Geza Halasz, Valeria Dall’Ospedale, Maddalena Conte, Valentina Parisi, Raffaele Galiero, Ferdinando Carlo Sasso, Giuseppe Fiorentino, Vincenzo Russo

**Affiliations:** 1 Cardiology Unit, Monaldi Hospital - A.O.R.N. Dei Colli, Naples, Italy; 2 Department of Advanced Medical and Surgical Sciences, University of Campania Luigi Vanvitelli, Naples, Italy; 3 Sub-intensive Care Unit and Respiratory Pathophysiology Department, Cotugno Hospital - A.O.R.N. Dei Colli, Naples, Italy; 4 First Division of Infectious Diseases, Cotugno Hospital - A.O.R.N. Dei Colli, Naples, Italy; 5 Respiratory Infectious Diseases Unit, Cotugno Hospital - A.O.R.N. Dei Colli, Naples, Italy; 6 Third Division of Infectious Diseases, Cotugno Hospital - A.O.R.N. Dei Colli, Naples, Italy; 7 Cardiology Department, Guglielmo Da Saliceto Hospital, Piacenza, Italy; 8 Cardiology Department, University of Parma, Parma, Italy; 9 Department of Translational Medical Sciences, University of Naples Federico II, Naples, Italy; 10 Division of Cardiology, Department of Medical Translational Sciences, University of Campania Luigi Vanvitelli, Naples, Italy

**Keywords:** Bradycardia, COVID-19, Pharmacovigilance, Remdesivir, Mortality, ARDS

## Abstract

**Introduction:**

Remdesivir exerts positive effects on clinical improvement, even though it seems not to affect mortality among COVID-19 patients; moreover, it was associated with the occurence of marked bradycardia.

**Methods:**

We retrospectively evaluated 989 consecutive patients with non-severe COVID-19 (SpO_2_ ≥ 94% on room air) admitted from October 2020 to July 2021 at five Italian hospitals. Propensity score matching allowed to obtain a comparable control group. Primary endpoints were bradycardia onset (heart rate < 50 bpm), acute respiratory distress syndrome (ARDS) in need of intubation and mortality.

**Results:**

A total of 200 patients (20.2%) received remdesivir, while 789 standard of care (79.8%). In the matched cohorts, severe ARDS in need of intubation was experienced by 70 patients (17.5%), significantly higher in the control group (68% vs. 31%; *p* < 0.0001). Conversely, bradycardia, experienced by 53 patients (12%), was significantly higher in the remdesivir subgroup (20% vs. 1.1%; *p* < 0.0001). During follow-up, all-cause mortality was 15% (*N* = 62), significantly higher in the control group (76% vs. 24%; log-rank *p* < 0.0001), as shown at the Kaplan–Meier (KM) analysis. KM furthermore showed a significantly higher risk of severe ARDS in need of intubation among controls (log-rank *p* < 0.001), while an increased risk of bradycardia onset in the remdesivir group (log-rank *p* < 0.001). Multivariable logistic regression showed a protective role of remdesivir for both ARDS in need of intubation (OR 0.50, 95%CI 0.29–0.85; *p* = 0.01) and mortality (OR 0.18, 95%CI 0.09–0.39; *p* < 0.0001).

**Conclusions:**

Remdesivir treatment emerged as associated with reduced risk of severe acute respiratory distress syndrome in need of intubation and mortality. Remdesivir-induced bradycardia was not associated with worse outcome.

## Introduction

Remdesivir (GS-5734) is approved by the European Medicine Agency (EMA) for patients with SARS-CoV-2 pneumonia requiring non-invasive supplemental oxygen at the beginning of the treatment, or for subjects who do not require supplemental oxygen, but are at increased risk of progressing to severe coronavirus disease 19 (COVID-19). Both clinical trials and observational studies showed that remdesivir exerts positive effects on clinical improvement, even though it does not seem to affect mortality [1]. Moreover, the occurrence of marked sinus bradycardia has been associated with remdesivir administration [2]. We aimed to investigate the association between remdesivir and the risk of bradycardia, acute respiratory distress syndrome (ARDS) in need of intubation, and mortality among non-severe COVID-19 patients hospitalized in medicine wards.

## Materials and methods

### Study population

We retrospectively evaluated a cohort of 1018 consecutive patients with non-severe COVID-19 admitted from October 2020 to July 2021 at medicine wards of five Italian hospitals. Among these, 29 were excluded due to incomplete data. COVID-19 diagnosis was initially based on the World Health Organization criteria and all subjects were later confirmed by real-time reverse transcriptase–polymerase chain reaction analysis of throat swab specimens. Non-severe COVID-19 was defined as evidence of lower respiratory disease during clinical assessment or imaging and oxygen saturation measured by pulse oximetry (SpO_2_) ≥ 94% on room air. The study population was divided according to the use of remdesivir into two groups. The discontinuation of remdesivir during the hospitalization was considered an exclusion criterion. This study was conducted according to the Declaration of Helsinki and approved by the institutional ethics committees. The requirement for informed consent from individual patients was waived due to the observational retrospective design of this study. Information on patient baseline characteristics, ARDS in need of intubation, bradycardia, and in-hospital mortality were prospectively collected and recorded on an electronic datasheet. ARDS diagnosis was defined according to the Berlin definition [3]. The severe form of ARDS based on the degree of hypoxemia was diagnosed when the ratio between arterial oxygen tension (PaO_2_) and the fraction of inspired oxygen (FIO_2_) was ≤ 100 mm Hg with positive end-expiratory pressure (PEEP) ≥ 5 cm H_2_O. Bradycardia was defined as heart rate < 50 bpm recorded at least one twelve-leads electrocardiogram and confirmed by single-lead continuous ECG recording.

### Statistical analysis

The Kolmogorov–Smirnov normality test was used to analyze data normality. Continuous variables were reported using the mean and standard deviation. Categorical variables were indicated as frequency counts and percentages. Baseline characteristics between remdesivir and no-remdesivir groups were compared by the *t* test for continuous variables and the chi-squared test for categorical variables. Nearest neighbour propensity score matching (PSM) with 1:1 ratio was used to minimize bias between groups. The variables included in the propensity score were age, male sex, obesity, hypertension, diabetes mellitus, chronic obstructive pulmonary disease, coronary artery disease, dilated cardiomyopathy, chronic kidney disease, and non-invasive ventilation.

The incidence of outcome events was calculated as cumulative incidence. Furthermore, clinical event recurrence-free rates in the study groups during follow-up were evaluated with the Kaplan–Meier method and compared with the log-rank test. The unadjusted (univariable) and adjusted (multivariable) odds ratios (OR) both for ARDS in need of intubation and in-hospital mortality were calculated using logistic regression models and presented as OR with their 95% confidence intervals (CI). Variables with a *p* ≤ 0.05 in univariate analysis were included in the multivariate regression model to correct for the interference of confounding factors, thus identifying the independent prognostic factors for the outcomes of interest. We considered statistically significant all *p* values less than 5%. All statistical analyses were performed using RStudio (RStudio Team (2016). RStudio: Integrated Development for R. RStudio, Inc., Boston, MA URL http://www.rstudio.com/).

## Results

### Study population baseline characteristics

A total of 989 patients with laboratory-confirmed non-severe COVID-19 (mean age 70 ± 11 years; male 72.3%) were included in the present analysis; among them, 200 patients (20.2%) received remdesivir at the recommended dosage (single loading dose of remdesivir 200 mg administered by intravenous infusion followed by 100 mg once daily for 4 days) within 10 days from symptoms onset. Since the two groups (taking or not taking remdesivir) were different for some baseline clinical characteristics, a propensity score matching cohort including 200 patients not taking remdesivir with balanced baseline characteristics was identified. All patients were treated with azithromycin 500 mg daily for 6 days, dexamethasone 8 to 16 mg per day depending on the severity of the COVID-19 pneumonia, and heparin at prophylactic dose (except in case of need of oral anticoagulants). The baseline characteristics of the study population, before and after matching, are summarized in Table 1.

**Table 1:** Characteristics of the study population before and after matching

**Variables**	**Remdesivir group** *n*: 200	**Control group** *n*: 789	***p* value**	**Matched control group** *n*: 200	***p* value**
Age, years	64.32 11.6	71.2 12.5	0.0001	64.4 14.1	0.95
Male, *n* (%)	148 (74%)	543 (69%)	0.17	139 (69%)	0.32
Obesity, *n* (%)	39 (19.5%)	55 (7%)	0.0001	39 (19.5%)	0.99
COPD, *n* (%)	20 (10%)	174 (22%)	0.0001	27 (13%)	0.27
Non-permanent AF, *n* (%)	9 (4.5%)	95 (12%)	0.50	15 (7.5%)	0.20
Permanent AF, *n* (%)	0 (0%)	24 (3%)	0.002	0 (0%)	0.99
Diabetes mellitus, *n* (%)	39 (19%)	205 (26%)	0.04	37 (18%)	0.8
Hepatopathy, *n* (%)	5 (2.5%)	24 (3%)	0.70	11 (5.5%)	0.13
Arterial hypertension, *n* (%)	112 (56%)	536 (68%)	0.0014	117 (58%)	0.61
CAD, *n* (%)	32 (16%)	189 (24%)	0.01	29 (14%)	0.68
DCM, *n* (%)	6 (3%)	87 (11%)	0.0005	2 (1%)	0.15
CKD, *n* (%)	13 (6.5%)	102 (13%)	0.01	23 (11.5%)	0.08
Heart rate, bpm	82.84 16.03	77.3 ± 8.2	0.001	85.03 15.93	0.17
First-degree AV block	10 (5%)	87 (11%)	0.01	8 (4%)	0.63
LBBB, *n* (%)	1 (0.5%)	16 (2%)	0.14	2 (1%)	0.56
RBBB, *n* (%)	25 (12%)	142 (18%)	0.04	17 (8.5%)	0.19
Correct QT, ms	412.43 42.6	422.55 34.7	0.005	403.83 29.23	0.12
ACE-I/ARBs, *n* (%)	70 (35%)	623 (79%)	0.0001	65 (32%)	0.59
Beta blockers, *n* (%)	53 (26%)	260 (33%)	0.06	61 (30%)	0.38
Amiodarone, *n* (%)	2 (1%)	32 (4%)	0.036	4 (2%)	0.41
Class IC AAR, *n* (%)	12 (6%)	91 (11.6%)	0.02	5 (2.5%)	0.08
Ca channel blockers,* n* (%)	38 (19%)	213 (27%)	0.02	36 (18%)	0.81
Digitalis drugs, *n* (%)	2 (1%)	16 (2%)	0.34	1 (0.5%)	0.56
Ivabradine, *n* (%)	3 (1.5%)	24 (3%)	0.24	1 (0.5%)	0.31
Antiplatelets, *n* (%)	43 (21%)	184 (23%)	0.54	32 (16%)	0.23
Anticoagulants, *n* (%)	12 (6%)	102 (13%)	0.006	11 (5.5%)	0.83
Statins, *n* (%)	37 (18%)	252 (32%)	0.0001	31 (15%)	0.42

*COPD* chronic obstructive pulmonary disease, *AF* atrial fibrillation, *CAD* coronary artery disease, *DCM* dilated cardiomyopathy, *CKD* chronic kidney disease, *AV* atrio ventricular, *LBBB* left bundle branch block, *RBBB* right bundle branch block, *ACE* angiotensin-converting enzyme, *ARBS* angiotensin II receptor blockers, *AAD* antiarrhythmics drugs

### Clinical outcome

**Fig. 1:**
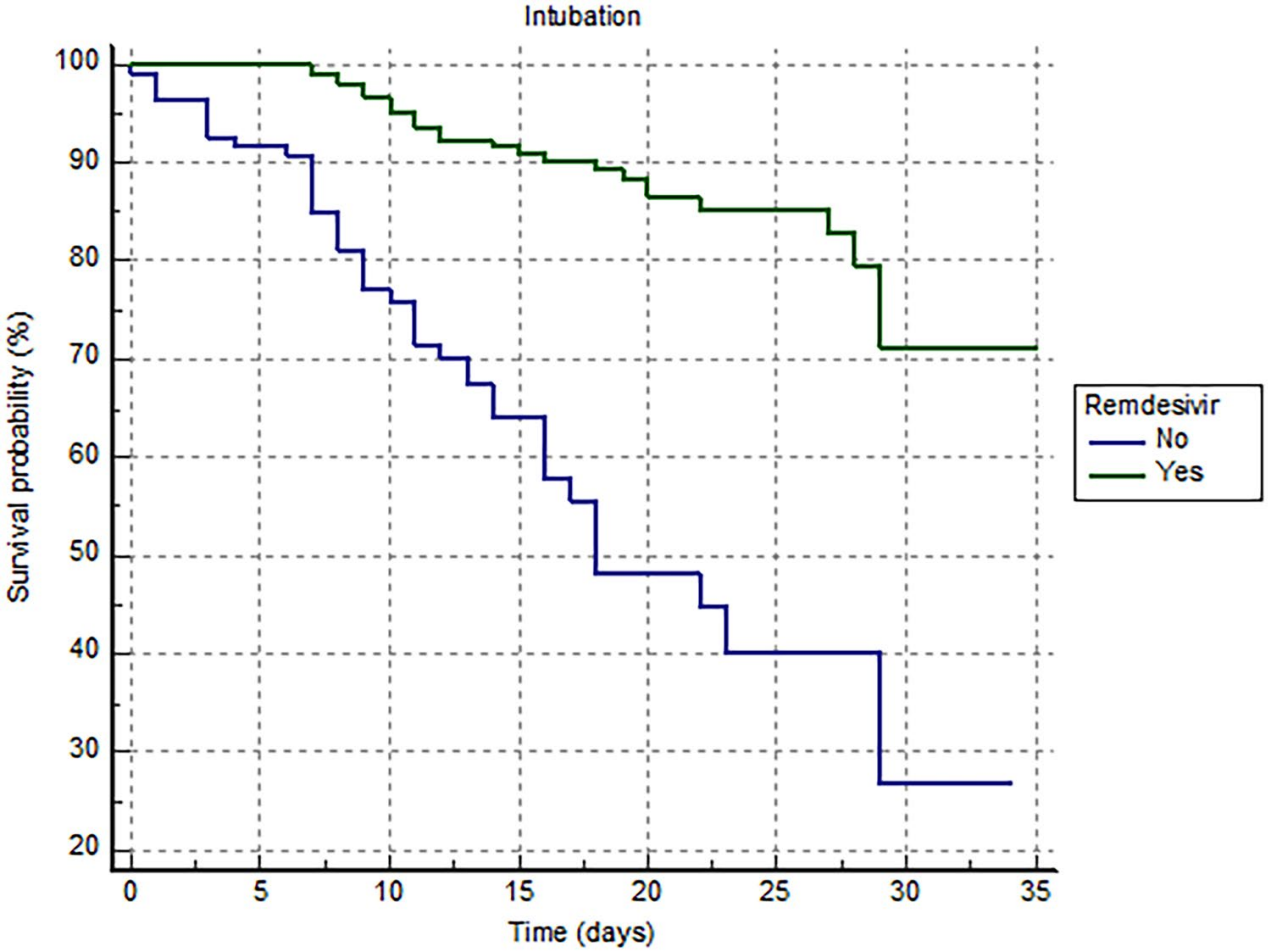
Kaplan–Meier survival analysis estimating the risk of ARDS in need of intubation among patients treated or not with remdesivir

In the matched cohorts, severe ARDS in need of intubation was experienced by 70 patients (17.5%): 22 patients (31%) in the remdesivir group and 48 patients (68%) in control group (*p* < 0.0001). The Kaplan–Meier analysis shows a significantly higher risk of severe ARDS in need of intubation in the control group compared with the remdesivir group (HR 0.50 [0.31–0.81]; log-rank *p* < 0.001) (Fig. 1).

**Fig. 2:**
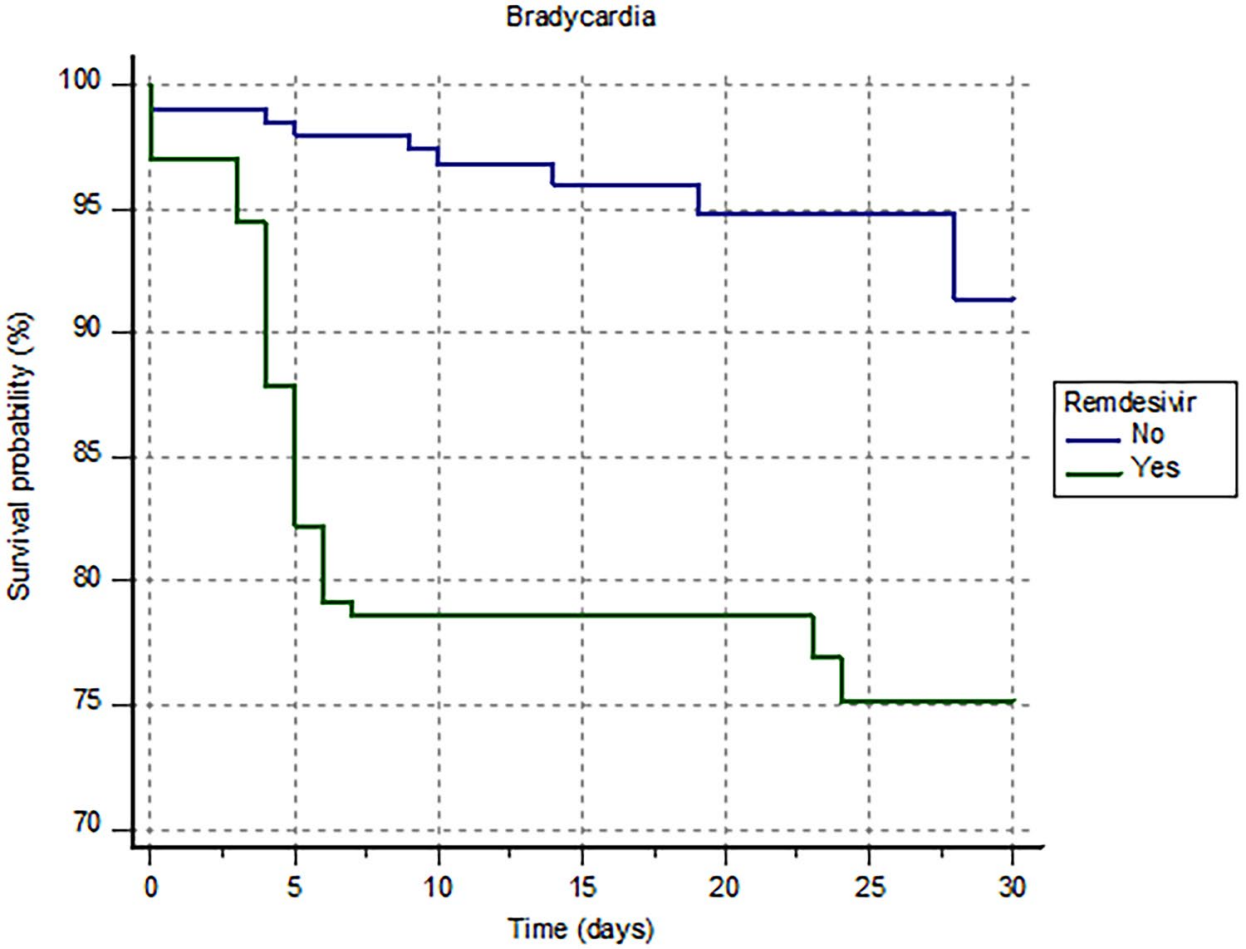
Kaplan–Meier survival analysis estimating the risk of bradycardia in patients treated or not with remdesivir

Forty-nine patients experienced bradycardia (12%): 40 patients (20%) in the remdesivir group and 9 patient (18%) in the control group (*p* < 0.0001). The Kaplan–Meier analysis shows a significantly higher risk of bradycardia in the remdesivir group compared with the control group (HR 5.16 [2.50–10.60]; log-rank *p* < 0.001) (Fig. 2).

**Fig. 3:**
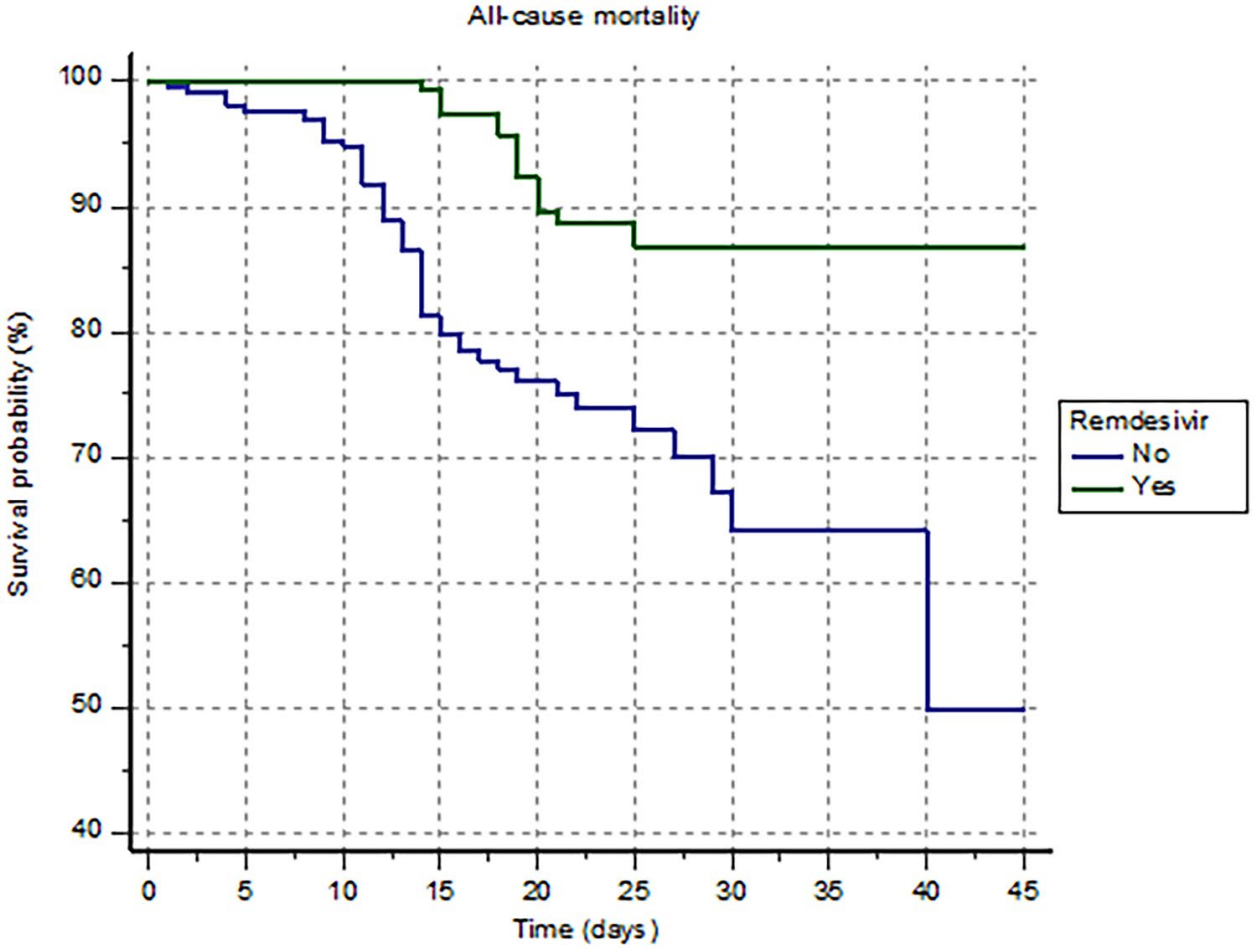
Kaplan–Meier survival analysis estimating the risk of all-cause mortality in patients treated or not with remdesivir

During the follow-up, 62 patients died for any cause (15%): 15 patients (24%) in the remdesivir group and 47 patient (76%) in the control group (*p* < 0.0001). The Kaplan–Meier analysis shows a significantly higher risk of all-cause death in the control group compared with the remdesivir group (HR 0.29 [0.16 to 0.51]; log-rank *p* < 0.001) (Fig. 3).

**Fig. 4:**
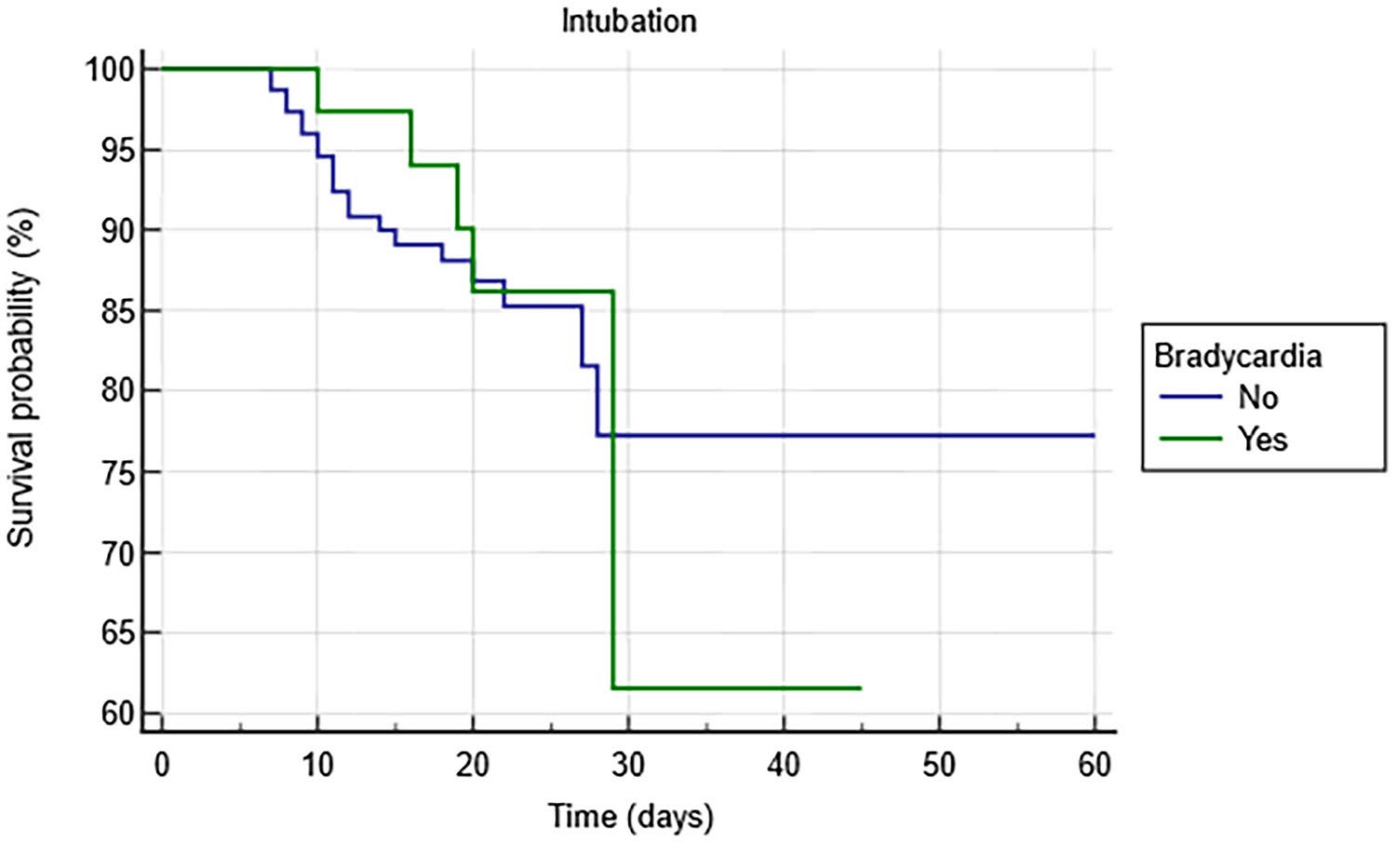
Kaplan–Meier survival analysis estimating the risk of ARDS in need of intubation among patients with remdesivir-induced bradycardia

**Fig. 5:**
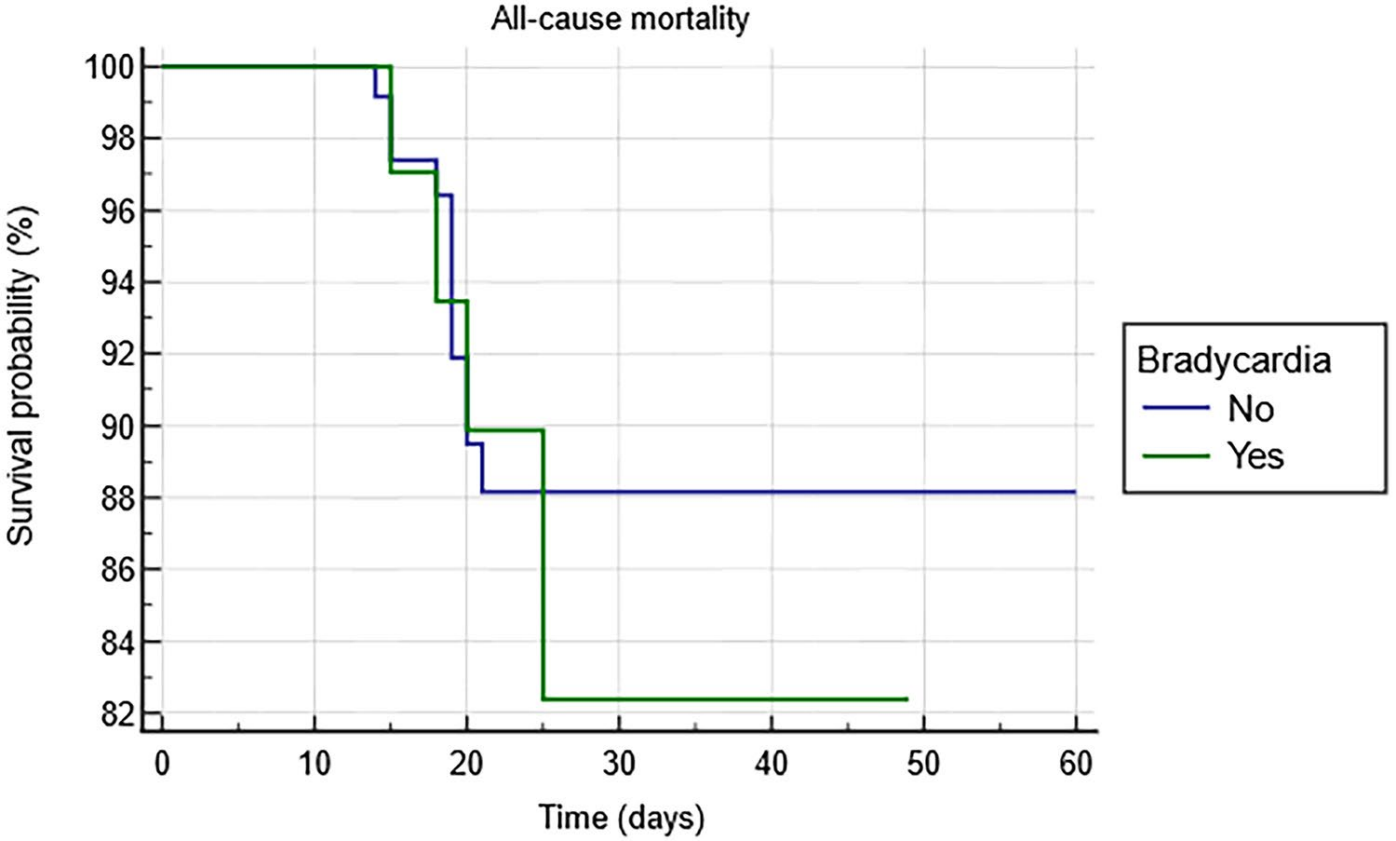
Kaplan–Meier survival analysis estimating the risk of all-cause mortality among patients with remdesivir-induced bradycardia

Among the remdesivir group, 40 patients experienced bradycardia. Patients on remdesivir with bradycardia showed similar incidence of ARDS in need of intubation (10% vs 11.3%; *p* = 0.89) and all-cause mortality (10% vs 6.9%; *p* = 0.65) compared to others (Table 2). The Kaplan–Meier analysis shows a non-significantly increased risk of both ARDS in need of intubation (HR 0.94 [0.38–2.35]; log-rank *p* = 0.895) (Fig. 4) and all-cause mortality in patients experiencing remdesivir-induced bradycardia (HR 1.17 [0.37–3.69]; log-rank *p* = 0.782) (Fig. 5).

**Table 2:** Clinical outcome of remdesivir patients with and without bradycardia

	**Remdesivir bradycardia** *n:* 40	**Remdesivir no-bradycardia** *n*: 160	***p* value**
ARDS in need of intubation	4 (10%)	18 (11.3%)	0.89
All-cause mortality	4 (10%)	11 (6.9%)	0.65

*ARDS* acute respiratory distress syndrome

### Univariate and multivariate analysis for the outcome of interests

Among matched population, male gender (OR 2.44 [1.22–4.88]; *p* = 0.01) was independently associated with severe ARDS in need of intubation; in contrast, remdesivir treatment significantly reduced this probability (OR 0.50 [0.29–0.85]; *p* = 0.01). Age (OR 1.08 [1.04–1.11]; *p* < 0.0001), obesity (OR 4.33 [1.68–11.13]; *p* = 0.002), and diabetes mellitus (OR 2.06 [1.00–4.22]; *p* = 0.048) were independently associated with in-hospital mortality; conversely, remdesivir treatment was protective (OR 0.18 [0.09–0.39]; *p* < 0.0001).

## Discussion

The main results of the present study are the following: remdesivir treatment was associated with a reduced risk to develop severe ARDS in need of intubation and in-hospital mortality. The increased risk of remdesivir-induced bradycardia seems to be not associated with worst outcome.

Among our real-world population including patients with non-severe COVID-19, remdesivir reduced the risk of severe ARDS in need of intubation by 50%; this finding was consistent with results from a Canadian randomized control trial, including 1884 patients randomized to receive remdesivir, which showed a reduction by 47% for the risk of mechanical ventilation [4]. A recent Cochrane systematic review, including five RCTs with 3886 participants randomized to receive remdesivir, concluded that remdesivir may decrease the risk of new need for invasive mechanical ventilation by 44% within 28 days when compared to placebo or standard care [5]. Remdesivir treatment benefit seems to be linked to the prevention of disease progression, suggesting an added value for patients with a less severe disease or at increased risk.

Regarding in-hospital mortality, the effects of remdesivir among hospitalized COVID-19 patients are still debated. Lee et al. in a recent meta-analysis including 10,751 patients from 8 randomized control trials showed a high probability, about 94%, that remdesivir reduces mortality for non-ventilated patients with COVID-19 requiring supplemental oxygen therapy [6]; by contrast, two previous meta-analyses did not show this result [7, 8]. These contrasting results may be explained by baseline different clinical characteristics, including age and disease severity, across the trials’ populations; moreover, it should be noted that in the early phase of the COVID-19 pandemic, patients were less likely to be vaccinated, as well as to receive treatment to reduce adverse outcomes (steroids, anticoagulation, monoclonal antibodies, and immunomodulatory therapies). In our study population, we showed that remdesivir treatment was associated with a reduction by 82% of in-hospital mortality compared to a PSM control group. The beneficial effect of remdesivir compared to standard care on morality has been previously showed only in the subgroup with low‐flow oxygen at baseline [9]. A recent matched case–control study, analyzing the use of remdesivir in severe and critical hospitalized patients, showed a survival benefit in comparison with controls, limited to patients on low-flow oxygen support; in contrast, the survival benefit was not present among patients who received the drug while on high-flow oxygen therapy or mechanical ventilation [10]. It is possible that the improvement in mortality outcome, at least partially, derives from the reduced disease progression requiring more invasive ventilation, which have been previously associated with worse clinical outcome [11].

Differently from previous reports which showed an overall incidence of sinus bradycardia following remdesivir ranging from 47 to 74% [12, 13], we reported a low prevalence of remdesivir-induced bradycardia, about 20%. These differences may be explained by the different definitions (heart rate < 60 beats/min or < 50 beats/min) and diagnoses (telemetry or ECG) across the studies [12, 13].

A recent large cohort study showed that bradycardia occurs up to 16.8% of severe/critical patients on day 5 of remdesivir use; it might reflect more favorable disease course and has a substantial potential for improving prognostication of patients with COVID-19 [14].

Remdesivir-related incident sinus bradycardia might be due to its status of nucleoside analog that resembles ATP, justifying its negative reversible chronotropic effect on sinus node cells [15, 16]. According to our findings, the incident sinus bradycardia following remdesivir administration did not seem to impact on patients’ prognosis in terms of intensive care unit admission and in-hospital mortality. Our results, despite the limitation of potential confounding factors, support a safe use of remdesivir among patients with COVID-19 in a real-world setting.

## Limitations

The retrospective nature of this study represents a bias due to the lack of randomization; however, the multicenter design, the relatively high sample size, and PSM might have overcome this issue. Since our study cohort was not continuously electrocardiography monitored, we cannot exclude the undetected short episodes of bradycardia; however, it is unlikely that an intermittent bradycardia pattern could affect both clinical decision-making and outcome. The analysis on mortality in patients on remdesivir with and without bradycardia is underpowered; however, it is similar to previous findings [17]. Finally, we have evaluated non-severe COVID-19 patients; thus, the results of our analysis are not generalized to more severe patients.

## Conclusions

In our propensity score matching analysis including patients with non-severe COVID-19 hospitalized at medicine wards, remdesivir treatment was associated with reduced risk of severe acute respiratory distress syndrome in need of intubation and in-hospital mortality. The remdesivir-induced bradycardia was not associated with worst outcome.

## Data Availability

The data that support the findings of this study are available from the corresponding author, VR, upon reasonable request.
